# Food-Grade Synthesis of Maillard-Type Taste Enhancers Using Natural Deep Eutectic Solvents (NADES)

**DOI:** 10.3390/molecules23020261

**Published:** 2018-01-28

**Authors:** Maximilian Kranz, Thomas Hofmann

**Affiliations:** Chair of Food Chemistry and Molecular and Sensory Science, Technical University of Munich, Lise-Meitner-Str. 34, D-85354 Freising, Germany; maximilian.kranz@tum.de

**Keywords:** Maillard reaction, Amadori rearrangement, food-grade synthesis, green chemistry, NADES, taste enhancers

## Abstract

The increasing demand for healthier food products, with reduced levels of table salt, sugar, and mono sodium glutamate, reinforce the need for novel taste enhancers prepared by means of food-grade kitchen-type chemistry. Although several taste modulating compounds have been discovered in processed foods, their Maillard-type ex food production is usually not exploited by industrial process reactions as the yields of target compounds typically do not exceed 1–2%. Natural deep eutectic solvents (NADES) are reported for the first time to significantly increase the yields of the taste enhancers 1-deoxy-d-fructosyl-*N*-β-alanyl-l-histidine (49% yield), *N*-(1-methyl-4-oxoimidazolidin-2-ylidene) aminopropionic acid (54% yield) and *N*^2^-(1-carboxyethyl) guanosine 5′-monophosphate (22% yield) at low temperature (80–100 °C) within a maximum reaction time of 2 h. Therefore, NADES open new avenues to a “next-generation culinary chemistry” overcoming the yield limitations of traditional Maillard chemistry approaches and enable a food-grade Maillard-type generation of flavor modulators.

## 1. Introduction

**Figure 1:**
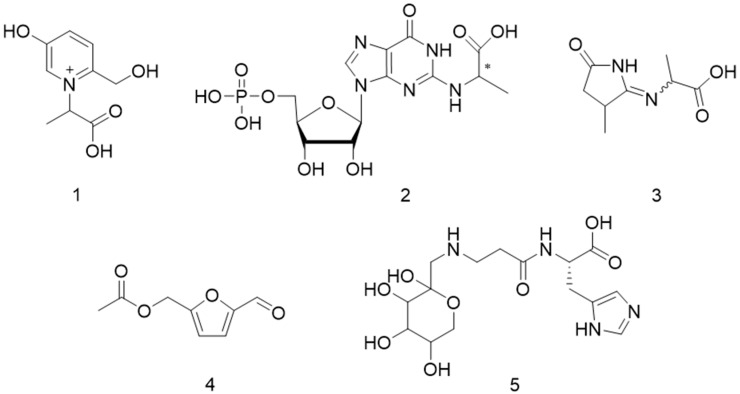
Chemical structures of Maillard-type taste enhancers alapyridaine (**1**), *N*^2^-(1-carboxyethyl) guanosine 5′-monophosphate (**2**), *N*-(1-methyl-4-oxoimidazolidin-2-ylidene) aminopropionic acid (**3**), 5-acetoxymethyl-2-furaldehyde (**4**), and 1-deoxy-d-fructosyl-*N*-β-alanyl-l-histidine (**5**).

The complex series of chemical reactions between reducing carbohydrates and free amino groups of proteins, peptides or amino acids that constitute the Maillard reaction is one of the most important sources for coloration of thermally treated foods, but also for the formation of aroma and taste [1,2]. Besides molecules that pronounce a characteristic odor in heated food, the Maillard reaction also brings molecules that contribute to taste sensations such as bitterness [3,4], cooling effects [5,6] or umami-like taste [7], respectively. By means of the so-called *sensomics* approach, the Maillard reaction has been demonstrated to generate taste modulating compounds which, although being taste- and odorless on their own, enhance or inhibit the perception of the basic taste modalities [8,9,10,11]. *N*-(1-Carboxyethyl)-6-(hydroxymethyl)pyridinium-3-ol inner salt (**1**; Figure 1), coined alapyridaine, has been reported as the first taste enhancing Maillard reaction product in beef broth, and was found to be thermally generated from alanine and glucose [12,13]. Functional enantiomer analysis revealed not only the (+)-(*S*)-isomer of alapyridaine to enhance the perceived sweetness of sugars, amino acids, and aspartame but also to decrease human recognition threshold concentrations of umami- and salty taste stimuli [12,13,14,15]. Moreover, derived from a Maillard-type carboxyethylation of guanosine 5′-monophosphate (5′-GMP) upon reaction with glyceraldehyde or dihydroxyacetone, respectively, the diastereomeric pair (*R*)- and (*S*)-*N*^2^-(1-carboxyethyl)-guanosine 5′-monophosphate (**2**) was identified as potent umami taste enhancers in processed yeast extracts [16]. Similarly, *N*-(1-methyl-4-oxoimidazolidin-2-ylidene) aminopropionic acid (**3**) was found to be generated upon carboxyethylation of creatine/creatinine upon reaction with glyceraldehyde and to induce an enhanced typical white-meaty, thick-sour and mouth-drying orosensation of an authentic stewed beef juice [17,18]. Furthermore, 5-acetoxymethyl-2-furaldehyde (**4**), discovered as a previously unknown sweet taste enhancer in Italian traditional balsamic vinegar, has been shown to be generated by esterification of the glucose/fructose degradation product 5-(hydroxymethyl)-2-furaldehyde (HMF) with acetic acid originating from acetic acid fermentation [19]. Very recently, 1-deoxy-d-fructosyl-*N*-β-alanyl-l-histidine (**5**) has been reported for the first time as the Amadori rearrangement reaction product of carnosine and glucose and was demonstrated to contribute to a white-meaty orosensation of meat-containing broths [20,21].

The increasing demand for healthier food products, with reduced levels of table salt, sugar, and mono sodium glutamate, and the growing aversion of alienated consumers towards non-natural chemicals added to foods reinforce the need of novel taste enhancers, either isolated from natural sources, or prepared by means of food-grade kitchen-type chemistry such as the Maillard reaction. Although thermal food processing has been demonstrated to generate taste modulators **1**–**5**, the industrial exploitation of such Maillard-type compounds is hampered by their low reaction yields, which typically do not exceed 1–2% when generated ex food in aqueous, low-moisture, or lipid reaction systems. Although lipid emulsions and self-assembled lipid structures have been developed as candidate reaction systems for an enhanced Maillard reaction chemistry [22,23,24], the low solubility of educts in the reaction system used, the hydrolytic instability of the reaction products, or the tendency to polymerize at elevated temperature, did yet enable the generation of flavor active compounds in suitable yields. Therefore, new food-grade reaction systems need to be designed to increase the yield of Maillard-type taste modulators using kitchen-type chemistry approaches. 

Next to aqueous solutions, lipid suspensions, and low-moisture mixtures of educts, the excellent dissolution properties of natural deep eutectic solvents (NADES), that have been reported for non or poorly water-soluble substances [25,26,27], make NADES a highly promising reaction medium for more efficient food-grade Maillard reactions. NADES are bio-based, low-vapor pressure liquids composed of two or more natural primary metabolites, i.e., organic acids, sugars, alcohols, amino acids or quaternary ammonium salts such as choline [25,26,28,29,30]. They are considered to form ionic bonds to give them liquid ionic crystal-like properties at room temperature due to the low symmetry of organic ions hindering crystallization and lower melting point, and/or hydrogen bonds to give them the typical properties of a deep eutectic solvent as mixtures of solid compounds form liquids due to a large depression of the melting point with the charge delocalization caused by hydrogen bonding [25]. Water molecules that participate in this supermolecular structure are strongly retained and cannot be evaporated afterwards [25,26]. The low production costs, low environmental and human toxicity, and the high thermal stability of the components typically used make NADES overcome use limitations of current ionic liquids in chemical industry [31,32]. Therefore, NADES have been used as extraction solvent, e.g., for the extraction of glycerol from biodiesel [33], flavonoids from plant material [34,35], or polyphenols from safflower and olive oil [36,37]. In alignment with the principles of green chemistry [38], moreover, NADES were successfully used as solvent mediums for enzymatic bioconversations [39,40] as well as in organic synthesis [41,42]. 

The objective of the present study was, therefore, to investigate for the first time the suitability of selected NADES systems to promote the ex food production of taste enhancers (**2**, **3**, and **5**) in comparison to current aqueous reaction systems, and to identify reaction conditions leading to increased yields of the target compounds.

## 2. Results and Discussion

### 2.1. Amadori Rearrangement 

1-Deoxy-d-fructosyl-*N*-β-alanyl-l-histidine (**5**) has been reported for the first time as the Amadori rearrangement reaction product of carnosine and glucose and was demonstrated to contribute to a white-meaty orosensation of meat-containing broths [20,21]. To better compare the yields of the Amadori product, generated in aqueous systems, with those in candidate NADES systems, first, the influence of the reaction time, temperature and the pH value was investigated in aqueous solution of carnosine and glucose.

**Figure 2:**
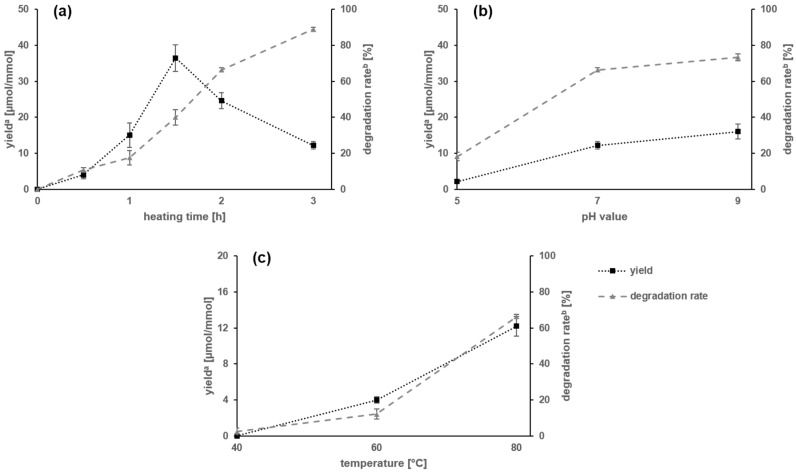
Stability and formation of the Amadori product 1-deoxy-d-fructosyl-*N*-β-alanyl-l-histidine (**5**) from glucose and carnosine in aqueous buffer solutions with (**a**) influence of the heating time on the formation and stability of **5** at pH 7.0 and 80 °C; (**b**) influence of the pH value on the formation and stability of **5** upon heating for 3 h at 80 °C; and (**c**) influence of the temperature on yield and stability of **5** upon heating for 3 h at pH 7.0. ^a^ Describes yields of the reaction as µmol of formed compound **5** per mmol of used carnosine. ^b^ Describes the percent degradation of **5** from an initial amount.

*Amadori product formation and stability in aqueous solutions.* Binary mixtures of carnosine and glucose were reacted in aqueous phosphate-buffered solutions and individual yields of the reaction product 1-deoxy-d-fructosyl-*N*-β-alanyl-l-histidine (**5**) were determined. To investigate the influence of the heating time on formation yield and stability of compound **5**, a first set of experiments was performed by reacting equimolar binary mixtures of carnosine and glucose or the target compound 1-deoxy-d-fructosyl-*N*-β-alanyl-l-histidine (**5**) at pH 7.0 at 80 °C for 0.5–3 h. After cooling, the Amadori rearrangement product was quantitated by means of LC-MS/MS and the formation and degradation rates, respectively, were calculated. The yield of 1-deoxy-d-fructosyl-*N*-β-alanyl-l-histidine (**5**) from carnosine and glucose was strongly affected by the reaction time, e.g., after running through a maximum of 36.5 µmol per mmol carnosine after 1.5 h, further prolongation of the reaction induced a decrease of **5** reaching a lower level of only 12.2 µmol/mmol after 3 h (Figure 2a). These data indicate the hydrolytic instability of the Amadori product and was confirmed by the drastic degradation observed when a purified sample of compound **5** was thermally treated with increasing time, e.g., 17% and 64% of the initial amount of the Amador production were degraded after 1 and 2 h of heating, respectively (Figure 2a). After 3 h, only 11% of the initial amount of **5** could be recovered, being well in line with the instability of the Amadori product that has been reported to be degraded to give 1- and 3-deoxyosones as transient reaction intermediates [2]. 

To investigate the influence of the pH value on the stability of the Amadori rearrangement product, a buffered aqueous solution of compound **5** was adjusted to pH 5.0, 7.0 and 9.0, respectively, and, then, heated for 3 h at 80 °C, followed by LC-MS/MS quantitation of **5**. In addition, equimolar mixtures of glucose and carnosine were reacted at pH 5.0, 7.0 and 9.0, respectively, to determine the yields of compound **5** generated. While the yield of the Amadori product reaction increased with increasing pH value to reach 2.1 (pH 5.0), 12.2 (pH 7.0), and 16.1 µmol/mmol (pH 9.0), the stability of compound **5** was increased at pH 5.0 with only 18% of the starting amount degraded and strongly decreased under alkaline conditions, e.g., 73% of **5** was degraded when reacted at pH 9.0 (Figure 2b), clearly demonstrating that the increased yield of the Amadori product formed from carnosine and glucose at higher pH value is counteracted by its decreased stability. 

In a third set of experiments, the carnosine/glucose reaction (pH 7.0, 3 h) was performed at 40, 60 and 80 °C, respectively, and the yields of target compound **5** determined by means of LC-MS/MS. Reaction at 40 °C did not lead to any Amadori product from glucose and carnosine and only a marginal degradation of 2% of compound **5** was observed (Figure 2c). With increasing temperature, the yield of the Amadori product **5** increased slightly from 4.0 (60 °C) to 12.2 µmol/mmol (80 °C) while, at the same time, stability of the target compound decreased, e.g., 12% and 64% of **5** were degraded after heating at 60 and 80 °C, respectively.

*Amadori product formation in NADES.* To investigate Amadori product formation in NADES, binary mixtures of glucose and carnosine were dissolved in different NADES systems (Table 1) and, then, reacted for 2 h at temperatures between 60 and 100 °C. In comparison, the same reaction was performed in aqueous buffer. With the exception of reactions in glucose/sucrose NADES, at which no additional glucose was utilized, the molar ratio of glucose toward carnosine was 2:1. After reaction, the Amadori product **5** was quantitatively determined by means of LC-MS/MS. 

**Figure 3:**
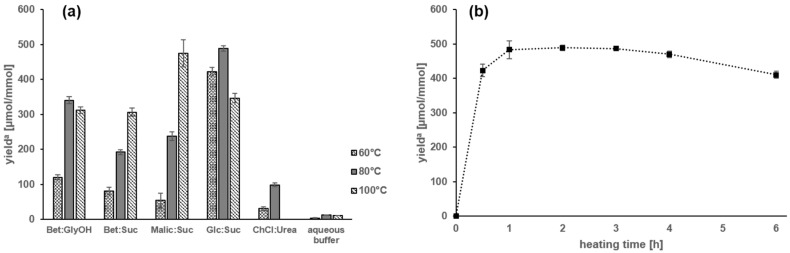
Yields of 1-deoxy-d-fructosyl-*N*-β-alanyl-l-histidine (**5**) from carnosine and glucose heated for 2 h in NADES (Table 1) made from betaine/glycerol (Bet:GlyOH), betaine/sucrose (Met:Suc), malic acid/sucrose (Malic:Suc), glucose/sucrose (Glc:Suc), and choline chloride/urea (ChCl:Urea), respectively, and aqueous buffer solutions (pH 7.0) with: (**a**) formation of **5** in NADES and aqueous buffer solutions as a function of temperature; and (**b**) influence of the heating time on yields of **5** in Glc:Suc NADES at 80 °C. ^a^ Yields of the reaction as µmol of formed compound **5** per mmol of used carnosine.

When the reaction was performed in NADES from betaine/glycerol and choline chloride/urea, respectively, yields of **5** were drastically increased running through a maximum at 80 °C, e.g., yields were 120.3 (60 °C), 340.4 (80 °C), and 312.7 µmol/mmol (100 °C) when the betaine/glycerol NADES was used (Figure 3a). In comparison, the glucose/carnosine reaction performed in aqueous solution afforded the Amadori product **5** in yields of maximal 12.2 µmol/mmol at 80 °C. Accordingly, temperatures above 80 °C were demonstrated to accelerate degradation in a greater extent than conversion was increased. In comparison to betaine/glycerol and choline chloride/urea, respectively, NADES made from betaine/sucrose and malic acid/sucrose delivered target compound **5** in highest yields when the reaction was performed at 100 °C, e.g., **5** was generated in yields of 306.4 and 474.9 µmol/mmol, respectively (Figure 3a). These results imply NADES from betaine/sucrose and malic acid/sucrose to stabilize the Amadori product. 

The highest yield of **5** (489.0 µmol/mmol) was found when carnosine was thermal treated in a NADES made from glucose/sucrose, which contains the reacting glucose as one of the NADES components (Figure 3a). High yields were achieved even at 60 (422.8 µmol/mmol) and 100 °C (346.6 µmol/mmol). As shown in Figure 3b, heating time seems to have only a minor effect on the yields of **5** produced in the NADES from glucose/sucrose, e.g., the yield of **5** reached a plateau of 423.3 µmol/mmol already after 30 min and increased marginally to 489.0 µmol/mmol after 2 h. This differs strongly to the aqueous buffer systems (Figure 2a) and makes the NADES from glucose/sucrose an ideal candidate for industrial Amadori product applications.

### 2.2. Carboxyethylation of Amino Compounds

Carboxyethylation of guanosine 5′-monophosphate (5′-GMP) and creatine/creatinine, respectively, upon Maillard-type reaction with C_3_-carbohydrates such as glyceraldehyde and its degradation product 2-oxopropanal, formed by β-elimination [43], was recently shown to lead to umami enhancers such as *N*^2^-(1-carboxyethyl)-5′-GMP (**2**) [16,44,45] in processed yeast extracts and glycated creatinine derivatives such as *N*-(1-methyl-4-oxoimidazolidin-2-ylidene) aminopropionic acid (**3**) inducing the authentic white-meaty, thick-sour and mouth-drying orosensation of stewed beef juice [17,18].

**Figure 4:**
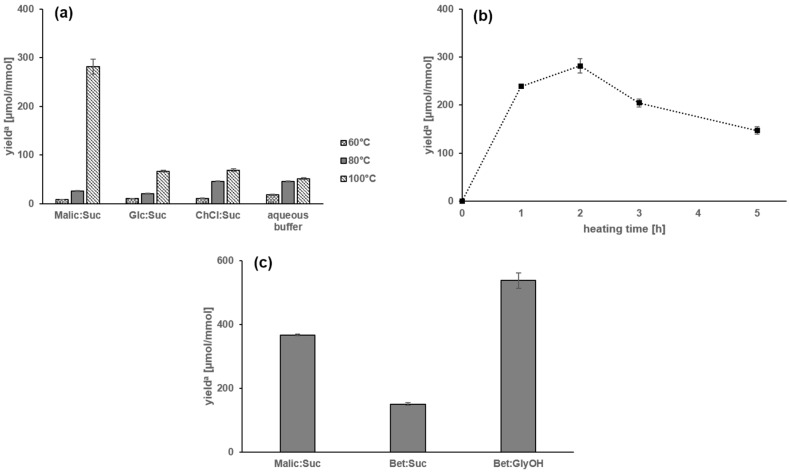
Yields of *N*-(1-methyl-4-oxoimidazolidin-2-ylidene)aminopropionic acid (**3**) from creatinine and glyceraldehyde with: (**a**) formation of **3** in NADES (Table 1) made from malic acid/sucrose (Malic:Suc), glucose/sucrose (Glc:Suc), and choline chloride/urea (ChCl:Urea), and in aqueous buffer solution (pH 7.0) as a function of temperature; (**b**) influence of the heating time on yields of **3** from creatine and glyceraldehyde (1:2) in NADES from malic acid/sucrose at 100 °C; and (**c**) formation of **3** from creatine and glyceraldehyde (1:2) in NADES (Table 1) made from malic acid/sucrose (Malic:Suc), betain/sucrose (Bet:Suc), and betain/glycerol (Bet:GlyOH) at 100 °C. ^a^ Yield of the reaction as µmol of formed compound **3** per mmol of used creatinine.

*Formation of N-(1-methyl-4-oxoimidazolidin-2-ylidene) aminopropionic acid* (**3**). Binary equimolar mixtures of creatinine and glyceraldehyde were reacted at 60, 80, and 100 °C for 2 h in different NADES, namely malic acid/sucrose, glucose/sucrose, and choline chloride/urea (Table 1) and, for comparison, in aqueous solution (pH 7.0) and the yield of *N*-(1-methyl-4-oxoimidazolidin-2-ylidene) aminopropionic acid (**3**) was determined by means of LC-MS/MS. As shown in Figure 4a, yields of compound **3** increased in aqueous solutions with increasing temperatures from 18.4 (60 °C) and 45.4 (80 °C) to 51.3 µmol/mmol (100 °C). While the reaction at 60 °C resulted in low conversion rates of less than 10 µmol/mmol in all NADES systems tested, elevated temperature of 80 and 100 °C revealed increased yields of 46.0 and 69.0 µmol/mmol in choline chloride/sucrose. Intriguingly, the individual NADES systems strongly affected the yields of the taste enhancer **3**. In particular, the reaction performed in NADES from malic acid/sucrose revealed a rather high conversion rate of 281.8 µmol/mmol when compared to the glucose/sucrose (66.9 µmol/mmol) or the choline chloride/sucrose system (69.0 µmol/mmol). 

To investigate the influence of reaction time, creatine and glyceraldehyde were reacted in a ratio of 1:2 in NADES from malic acid/sucrose at 100 °C (Figure 4b). The yield of **3** increased with increasing reaction time to reach 231.9 µmol/mmol after 1 h and, after running through a maximum of 281.8 µmol/mmol after 2 h, decreased again to a minimum of 147.0 µmol/mmol after 5 h. Finally, creatine and glyceraldehyde were reacted in a ratio of 1:2 in NADES from malic acid/sucrose, betaine/sucrose and betaine/glycerol, respectively, at 100 °C. Yields of **3** generated in NADES from malic acid/sucrose were 367.0 µmol/mmol and significantly lower in NADES from betaine/sucrose (150.1 µmol/mmol). However, the NADES from betaine/glycerol gave the high yield of 537.6 µmol/mmol (Figure 4c), which makes this system an interesting candidate for industrial use.

**Figure 5:**
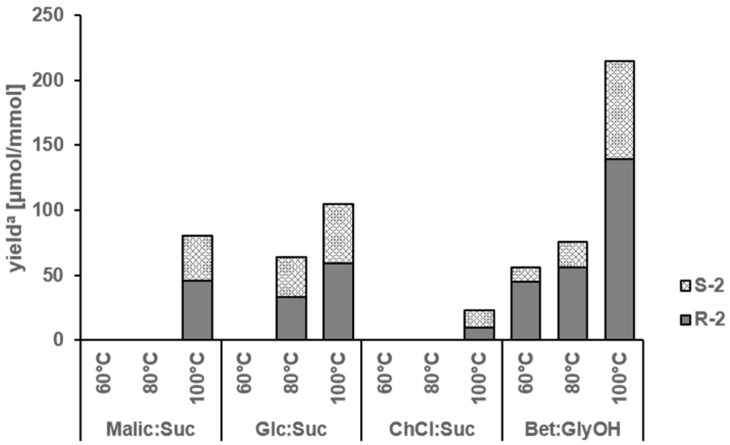
Yield of (*R*)- and (*S*)-*N*^2^-(1-carboxyethyl)guanosine 5′-monophosphate (**2**) from an equimolar mixture of glyceraldehyde and 5′-GMP in NADES (Table 1) made from betaine/glycerol (Bet:GlyOH), malic acid/sucrose (Malic:Suc), glucose/sucrose (Glc:Suc), and choline chloride/urea (ChCl:Urea) as a function of temperature. ^a^ Yield of the reaction as µmol of formed compound **2** (R-2, S-2 and their sum) per mmol of used 5′-GMP.

*Formation of N^2^-(1-carboxyethyl)guanosine 5′-monophosphate* (**2**). As the reaction leading to *N*^2^-(1-carboxyethyl)guanosine 5′-monophosphate (**2**) from 5′-GMP and glyceraldehyde is expected to be similar to the one giving rise to *N*-(1-methyl-4-oxoimidazolidin-2-ylidene)aminopropionic acid (**3**) from creatinine and glyceraldehyde, and compound **2** has been reported to need a long reaction time of at least 24 h at 70 °C to be formed in substantial amounts [45], a similar design of experiments was applied to the formation of the umami enhancer **2**. Binary equimolar mixtures of 5′-GMP and glyceraldehyde were reacted at 60, 80, and 100 °C for 2 h in different NADES, namely malic acid/sucrose, glucose/sucrose, choline chloride/urea, and betaine/glycerol (Table 1), and the yield of (*R*)- and (*S*)-*N*^2^-(1-carboxyethyl)guanosine 5′-monophosphate (**2**) was determined by means of LC-MS/MS (Figure 5). The highest yields of **2** (sum of (*R*)-**2** and (*S*)-**2**) were found when the reaction was performed at 100 °C in NADES from betaine/glycerol (215.3 µmol/mmol), followed by glucose/sucrose (104.8 µmol/mmol) and malic acid/sucrose (80.0 µmol/mmol). In comparison, choline chloride/sucrose did not afford **2** in high yields (22.7 µmol/mmol). While lower yields of **2** were found upon decreasing the temperature from 100 to 80 °C, no detectable amounts of **2** were formed at 60 °C with the exception of the betaine/glycerol system, which still afforded the target compound in yields of 56.1 µmol/mmol.

The distribution of the diastereomers (*R*)-**2** and (*S*)-**2** was about 1:1 and nearly identical in the NADES systems malic acid/sucrose, glucose/sucrose, and choline chloride/urea (Figure 5). Intriguingly, the (*R*)-enantiomer of **2** seems to be slightly favored when the reaction was performed in NADES from betaine/glycerol at low temperatures, e.g., 80%, 74%, and 65% of (*R*)-**2** was found after reaction at 60, 80, and 100 °C.

Following the *sensomics*-based discovery of new taste modulating compounds in processed foods [16,17,18,44,45], the data shown here open new avenues to a “next-generation culinary chemistry“ approach which utilizes the potential of natural deep eutectic solvents (NADES) to overcome the yield limitations of traditional Maillard chemistry approaches and enables a *food-grade* Maillard-type generation of flavor modulators, such as *N*^2^-(1-carboxyethyl) guanosine 5′-monophosphate (**2**), *N*-(1-methyl-4-oxoimidazolidin-2-ylidene) aminopropionic acid (**3**), and 1-deoxy-d-fructosyl-*N*-β-alanyl-l-histidine (**5**). In addition, the accelerated generation of some Maillard reaction products in the presence of NADES may explain the unexpected high levels of 0.1–2% (dry weight) found for Amadori products in dried fruits and vegetables, such as *N*-(1-deoxy-d-fructos-1-yl)-l-asparagine [46] in heat-dried apricots, apples and asparagus, and *N*-(1-deoxy-d-fructos-1-yl)-l-glutamate [47] in sun-dried tomatoes, which may be considered as a “food-NADES” system of primary metabolites, such as organic acids, sugars, and amino acids, respectively. Likewise, the NADES systems do not only allow an ex food generation of taste modulators but might also, without further purification, be used directly as part of food ingredients to enable an enhanced flavor generation in situ during food processing. The high productivity of the NADES systems to form flavor-active target molecules even at low temperatures of 60 to 100 °C may be beneficial to prevent the generation of undesired process contaminants that are known to be formed under low-moisture conditions at higher temperatures (>170 °C) such as acrylamide [48], furans [49,50] and α-dicarbonyls [51]. This needs to be further investigated in future studies. 

## 3. Materials and Methods 

### 3.1. Chemicals

Ultrapure water for chromatographic separation was prepared with a Milli-Q Gradient A 10 system (Millipore, Schwalbach, Germany), and solvents were of HPLC grade quality (Baker J.T., Deventer, The Netherlands). All chemicals were purchased from Sigma Aldrich (Steinheim, Germany) or Fluka (Neu-Ulm, Germany). l-carnosine was obtained from Bachem (Bubendorf, Switzerland), and sucrose was purchased from Merck (Darmstadt, Germany). Stable isotope-labeled compounds were supplied by Euriso-Top (Saarbruecken, Germany). (*R*)- and (*S*)-*N*^2^-(1-carboxyethyl) guanosine 5′-monophosphate (**2**), [^13^C_3_]-labeled (*R*)- and (*S*)-**2**, and *N*-(1-methyl-4-oxoimidazolidin-2-ylidene) aminopropionic acid (**3**) were prepared, purified by chromatography and their chemical structure verified by LC-TOF-MS and 1D/2D-NMR as reported recently [16,17]. Natural ^13^C-abundant as well as ^13^C_6_-labeled 1-deoxy-d-fructosyl-*N*-β-alanyl-l-histidine (**5**) was prepared using a literature protocol, purified by chromatography and their chemical structure verified by LC-TOF-MS and 1D/2D-NMR [21].

### 3.2. Preparation of NADES

NADES were prepared as reported in the literature using the so-called heating method [25]. Compositions and molar ratios of the NADES are summarized in Table 1. Components were placed with calculated amounts of water in a glass vial, and heated at a temperature of 50 °C while agitating. After 2 h (Bet:GlyOH NADES) to 10 h (Malic:Suc NADES) of agitation, the NADES were cooled to room temperature and stored in a desiccator until use.

### 3.3. Experimental Setup

*Formation of 1-deoxy-*d*-fructosyl-N-*β*-alanyl-*l*-histidine* (**5**) *in aqueous buffered solutions*. A binary mixture of carnosine (1 mmol) and glucose (1 or 2 mmol) in aqueous Na_2_HPO_4_ buffer (0.5 mol/L; pH 5–9; 20 mL) was heated for 0.5 h to 3 h at temperatures between 40 and 100 °C in a closed vessel. The reaction was stopped by the addition of ice water, and the solution was made up to 100 mL with water.

*Formation of 1-deoxy-*d*-fructosyl-N-*β*-alanyl-*l*-histidine* (**5**) *in NADES*. A binary mixture of carnosine (0.44 mmol) and glucose (0.88 mmol) in NADES (Table 1, 8 g each) was heated for 0.5 to 6 h at temperatures between 60 and 100 °C. The reaction was stopped by the addition of ice water, and the solution was made up to 100 mL with water.

*Formation of N-(1-methyl-4-oxoimidazolidin-2-ylidene)aminopropionic acid* (**3**) *in aqueous buffered solutions*. A binary mixture of creatinine (1 mmol) and glyceraldehyde (1 mmol) in aqueous Na_2_HPO_4_ buffer (0.5 mol/L; pH 7; 10 mL) was heated for 2 h at temperatures between 60 and 100 °C in a closed vessel. The reaction was stopped by the addition of ice water, and the solution was made up to 100 mL with water.

*Formation of N-(1-methyl-4-oxoimidazolidin-2-ylidene)aminopropionic acid* (**3**) *in NADES*. A binary mixture of creatinine (1 mmol) and glyceraldehyde (1 mmol, 2 mmol, or 4 mmol) in NADES (Table 1, 8 g each) was heated for 2 to 5 h at temperatures between 60 and 100 °C in a closed vessel. The reaction was stopped by the addition of ice water, and the solution was made up to 100 mL with water.

*Formation of N^2^-(1-carboxyethyl)-guanosine 5′-monophosphate* (**2**) *in NADES*. A binary mixture of 5′-GMP (0.33 mmol) and glyceraldehyde (0.33 mmol) in NADES (Table 1, 8 g each) was heated for 2 h at temperatures between 60 and 100 °C in a closed vessel. The reaction was stopped by the addition of ice water, and the solution was made up to 100 mL with water.

### 3.4. High-Performance Liquid Chromatography–Mass Spectrometry (HPLC-MS/MS)

LC-MS/MS analysis was performed using a Dionex Ultimate 3000 HPLC-system connected to either an API 3200 MS/MS, or an API 4000 QTrap MS/MS device (both AB Sciex, Darmstadt, Germany) running in the positive or negative electrospray ionization (ESI^+^, ESI^−^) mode, respectively. Zero grade air served as nebulizer gas (45 psi), and as turbo gas for solvent drying (55 psi, 425 °C). Nitrogen served both as curtain gas (35 psi) and collision gas (8.7 × 10^−7^ psi). Both quadrupoles were set at unit resolution. ESI^+^ and ESI^−^ mass and product ion spectra were acquired with direct flow infusion. For ESI^+^, the ion spray voltage was set at +5500 V and −4500 V for ESI^−^. Energies for declustering potential (DP) entrance potential (EP), collision energy (CE), and cell exit potential (CXP) as well as MS/MS parameters for measuring in the MRM mode were optimized for each compound individually, detecting the fragmentation of molecular ions into specific product ions after collision with nitrogen. For instrumental control and data acquisition, Sciex Analyst software v1.6.2 (Sciex, Darmstadt, Germany) was used. Quantitative data are given as the mean of triplicates, assessed by using calibration curves that are calculated by plotting peak area ratios of the analyte to the internal standard versus the concentration ratios followed by linear regression.

*Quantitative Analysis of N^2^-(1-carboxyethyl)-guanosine 5′-monophosphate* (**2**). For the LC-MS/MS quantitation of (*R*)- and (*S*)-**2**, aliquots of the sample were diluted with water (1/250; *v*/*v*) and membrane filtered (0.45 µm) prior to analysis. Sample solutions (990 µL) were spiked with an internal standard solution (10 µL) of [^13^C_3_]-labeled (*R*)- and (*S*)-**2** (400 mg/L). Aliquots (2 µL) were directly injected into an API 4000 QTrap LC-MS/MS mass spectrometer (AB Sciex, Darmstadt, Germany), equipped with a Luna PFP column (150 × 2.00 mm, i.d., 3 µm, Phenomenex, Aschaffenburg, Germany) and guard column of the same type. Chromatography was performed with a flow rate of 0.2 mL/min and started with a mixture (97/3, *v*/*v*) of aqueous formic acid (0.1% in water, solvent B) and acidified acetonitrile (0.1% formic acid, solvent A). After 3 min of isocratic conditions, the content of solvent A was increased to 100% within 22 min and kept isocratic for 2 min. Within another 10 min, starting conditions were restored and kept for another 8 min. Analytes and stable-isotope labeled internal standards were analyzed in the negative electrospray ionization mode (ESI^−^), using optimized tuning parameters for each compound. Given in parentheses are the used mass transitions, declustering potential (DP in V), collision energy (CE in V), and cell exit potential (CXP in V): (*R*)-/(*S*)-*N*^2^-(1-carboxyethyl)-5′-GMP (*m*/*z* 434.1→336.0; −85/−30/−11), [^13^C_3_]-(R)-/[^13^C_3_]-(S)-*N*^2^-(1-carboxyethyl)-5′-GMP (*m*/*z* 437.1→339.0; −80/−28/−13).

*Quantitative Analysis of N-(1-methyl-4-oxoimidazolidin-2-ylidene)aminopropionic acid* (**3**). *N*-(1-methyl-4-oxoimidazolidin-2-ylidene)aminopropionic acid was quantified by means of LC-MS/MS analysis using [^2^H_3_]-creatinine as an internal standard. Prior to analysis, aliquots of the samples were diluted with water (1/500; *v*/*v*), membrane filtered (0.45 µm), and aliquots (990 µL) were spiked with an aliquot (10 µL) of a [^2^H_3_]-creatinine solution (500 mg/L). Aliquots (2 µL) were directly injected into an API 3200 LC-MS/MS mass spectrometer (AB Sciex, Darmstadt, Germany), equipped with a TSKgel Amide-80 column (150 × 2.00 mm, i.d., 3 µm, Tosoh Bioscience, Stuttgart, Germany) and guard column of the same type. Chromatography was performed with a mixture (95/5; *v*/*v*) of acetonitrile and aqueous ammonium acetate (100 mmol/L), adjusted to pH 3.0 with acetic acid (solvent A), and aqueous ammonium acetate (5 mmol/L), adjusted to pH 3.0 with acetic acid (solvent B) at a flow rate of 0.2 mL/min. Starting with a mixture of 75% A and 25% B for 2 min, the content of solvent B was increased to 40% within 1 min. Afterwards, these conditions were kept for 2 min, before the content of solvent B was increased to 100% within 1 min. These conditions were kept for 3 min, and finally adjusted to match the starting conditions within 1 min. Equilibration time was then another 4 min. Analyte and the internal standard were analyzed in the positive electrospray ionization mode (ESI^+^), using optimized tuning parameters for each compound. Given in parentheses are the used mass transitions, declustering potential (DP in V), collision energy (CE in V), and cell exit potential (CXP in V): *N*-(1-methyl-4-oxoimidazolidin-2-ylidene)aminopropionic acid (*m*/*z* 186.1→113.9; +31/+23/+4), [^2^H_3_]-creatinine (*m*/*z* 117.0→47.0; +51/+21/+2).

*Quantitative Analysis of 1-Deoxy-*d*-fructosyl-N-*β*-alanyl-*l*-histidine* (**5**). 1-Deoxy-d-fructosyl-*N*-β-alanyl-l-histidine was quantified by means of LC-MS/MS analysis. After dilution with water (1/500; *v*/*v*), the samples were membrane filtered (0.45 µm), aliquots (990 µL) were spiked with an aliquot (10 µL) of a [^13^C_6_]-1-deoxy-d-fructosyl-*N*-β-alanyl-l-histidine solution (150 mg/L). Aliquots of the sample (3 µL) were directly injected into an API 4000 QTrap LC-MS/MS mass spectrometer (AB Sciex, Darmstadt, Germany), equipped with a TSKgel Amide-80 column (150 × 2.00 mm, i.d., 3 µm, Tosoh Bioscience, Stuttgart, Germany) and guard column of the same type. Chromatography was performed with a mixture (95/5; *v*/*v*) of acetonitrile and aqueous ammonium acetate (100 mmol/L), adjusted to pH 3.2 with acetic acid (solvent A), and aqueous ammonium acetate (5 mmol/L), adjusted to pH 3.2 with acetic acid (solvent B) at a flow rate of 0.2 mL/min. Starting with a mixture of 80% A and 20% B for 5 min, the content of solvent B was increased to 100% within another 5 min. Afterwards, these conditions were kept for 7 min before the starting conditions of 80% A and 20% B were reassembled and kept for an additional 5 min. Analyte and stable-isotope labeled internal standard were analyzed in the positive electrospray ionization mode (ESI^+^) using optimized tuning parameters for each compound. Given in parentheses are the used mass transitions, declustering potential (DP in V), collision energy (CE in V), and cell exit potential (CXP in V): 1-deoxy-d-fructosyl-*N*-β-alanyl-l-histidine (*m*/*z* 389.2→305.0; +71/+25/+4), [^13^C_6_]-1-deoxy-d-fructosyl-*N*-β-alanyl-l-histidine (*m*/*z* 395.3→310.1; +81/+25/+10).

## Figures and Tables

**Table 1:** NADES used for this study, and respective abbreviations and molar ratios.

NADES		Sample Name	Ratio ^a^
Component 1	Component 2		
Choline chloride	Sucrose	ChCl:Suc	4:1:4
Choline chloride	Urea	ChCl:Urea	1:2:1
Malic acid	Sucrose	Malic:Suc	1:1:5
Glucose	Sucrose	Glc:Suc	1:1:9
Betaine	Sucrose	Bet:Suc	2:1:9
Betaine	Glycerol	Bet:GlyOH	1:2:2

^a^ Ratio describes the molar ratio according to the following scheme: component 1:component 2:water.
